# Pregnancy and oncologic outcomes of early stage low grade epithelial ovarian cancer after fertility sparing surgery: a retrospective study in one tertiary hospital of China

**DOI:** 10.1186/s13048-019-0520-6

**Published:** 2019-05-14

**Authors:** Jie Yin, Yongxue Wang, Ying Shan, Yan Li, Ying Jin, Lingya Pan

**Affiliations:** Department of Obstetrics and Gynecology, Peking Union Medical College Hospital, Chinese Academy of Medical Science and Peking Union Medical College, Beijing, China

**Keywords:** Epithelial ovarian cancer, Fertility preservation, Reproductive outcome, Oncologic outcomes

## Abstract

**Objective:**

To detected both pregnancy and oncologic outcomes early stage low grade epithelial ovarian cancer (EOC) in one tertiary hospital of China.

**Methods:**

Medical records of 40 EOC patients between January 2000 and December 2016 in Peking Union Medical College Hospital (PUMCH) were retrospectively reviewed.

**Results:**

All patients were IA/IC low grade EOC patients. Mean age was 28 years (ranged from 18 to 44 years). Among them, 47.5% had mucinous carcinoma, 22.5% clear cell carcinoma, 20% endometriod carcinoma, 10% serous carcinoma. 40% of patients were operated under laparoscopy. 67.5% of patients received chemo-therapy after surgery. The median follow up time was 54 months (ranged from 25 to 201 months). The 2 year and 5 year of disease free survival were respectively 95 and 87.5%. No death occurred. 27 patients (67.5%) were single or contraceptive. 13 patients (32.5%) tried to be pregnant, and 8 patients (61.5%) have successfully conceived spontaneously, including 1 fetus loss, 1 fetus death in uterus, 1 fetal anomaly and 5 live births (2 premature births and 3 term births). There was no tumor recurrence happened during pregnancy.

**Conclusions:**

Fertility sparing surgery was safe and pregnancy was encouraged to stage IA/IC low grade EOC patients.

## Introduction

Ovarian cancer is one of the most common malignancies of the female reproductive system in the world. The main pathological type is epithelial ovarian cancer (EOC). Around 10% of EOC is diagnosed in patients younger than 40 years. Preservation of fertility is very important for young patients of early EOC. The 5 years survival in stage I EOC ranges from 70~100%, which depend on tumor biology and postsurgical treatments [1]. The primary treatment of ovarian cancer consists of appropriate surgical staging and debulking surgery, followed in most patients by systemic chemotherapy. The complete surgical staging includes total abdominal hysterectomy (TAH) and bilateral salpingo-oophorectomy (BSO). However, for young patients who wish to preserve fertility, a unilateral salpingo-oophorectomy (USO) and comprehensive surgical staging may be adequate for select unilateral stage I tumors (FIGO stage IA and IC) [2–4]. For those with FIGO stage IB tumors who wish to maintain fertility, a BSO (preserving the uterus) and comprehensive surgical staging are needed. Careful surgical staging is critical for early EOC patients to rule out occult higher-stage disease, because nearly 30% of patients undergoing complete staging surgery are upstaged. Compared with conventional surgery, fertility sparing surgery (FSS) was not associated with increased risk of death in young women with stage I epithelial ovarian cancer [5]. According to many recent reports, the oncological outcomes of patients after fertility-sparing surgery are good, but their pregnancy outcomes are still unknown.

Since 2000, we performed fertility sparing surgery on low grade early stage epithelial ovarian cancer patients who wished to maintain fertility and could be followed up as required in Peking Union Medical College Hospital (PUMCH), Beijing, China. It is not known how many of those will finally become pregnant and have live births. Here, besides oncologic outcome, we extremely focused on their pregnancy outcome and pregnancy attempts.

## Patients and methods

Medical records of EOC patients who had undergone fertility sparing surgery between January 2000 and December 2016 in Peking Union Medical College Hospital (PUMCH) were reviewed retrospectively. This study was approved by the ethics committee of PUMCH. All patients who received fertility sparing surgery were included. All patients received comprehensive surgical staging surgery, including unilateral salpingo-oophorectomy, a complete exploration of the whole peritoneum with multiple biopsies, washing cytology, omentectomy, bilateral pelvic lymph node dissection, and para-aortic lymph node dissection. Patients were staged according to the International Federation of Gynecology and Obstetrics criteria (FIGO, 2013) [6], using macroscopic findings and histological analysis of specimens obtained during staging surgery. Pathology slides were all reviewed by one pathologist in PUMCH. Histological cell type and tumor differentiation were assessed according to the World Health Organization (WHO) criteria [7]. Patients of non-epithelial ovarian cancer and borderline tumors were excluded.Clear cell carcinoma or stage IC plus disease was the indication of post-operative chemo-therapy. Most of patients after fertility sparing surgery received carboplatin/cisplatin based chemo-therapies. And all of the patients could be followed up every 3 months for the first 3 years, every 6 months for the next 2 years, then once in a year. Pregnancy and oncologic outcomes were evaluated.

## Results

During January 2000 to December 2016, 40 patients of epithelial ovarian cancer supposed to early-stage received comprehensive fertility-sparing surgery at PUMCH. The baseline clinical data of all patients was shown in Table 1. All patients were low grade epithelial ovarian carcinoma. Median age at surgery was 28 years old (ranged from 18 to 44 years). 38 patients were early stage (FIGO stage IA or IC) epithelial ovarian cancer. Two patients were upstaged after staging surgery according to FIGO criteria. One was stage IIB (laparoscopic staging surgery) and have disease recurrence after 5 months. The other was stage IIIA1 (laparotomy staging surgery) endometriod type with para-aortic lymph-node micro metastasis. She rejected additional TAH and USO, and received chemotherapy with paclitaxel and carboplatin for 6 cycles. No disease recurrence was found after following up 5 years. The tumor differentiations of all 40 patients were low grade. The mucinous type of tumor was the most common (47.5%) histologic type. Then the next in sequence were clear cell (22.5%), endometriod (20%) and serous (10%). 40% of patients were operated under laparoscopy. Most of patients (67.5%) received chemo-therapy after surgery, including 5 stage IA patients who were all clear cell carcinoma. 7 patients with stage IC disease and 1 clear cell carcinoma with stage IA disease rejected post-operative chemo-therapy.

**Table 1:** Clinical oncological data of all 40 patients of epithelial ovarian cancer

	N (%)
Total	40
Age at surgery, years Median (range)	28 (18~44)
Follow up, months Median (range)	54 (25~201)
Histology	
Serous	4 (10%)
Mucinous	19 (47.5%)
Endometriod	8 (20%)
Clear cell	10 (22.5%)
FIGO stage	
IA	11 (27.5%)
IB	0
IC	27 (67.5%)
IIA	0
IIB	1 (2.5%)
IIC	0
IIIA	1 (2.5%)
IIIB	0
IIIC	0
Grading	
Low	40 (100%)
High	0
Type of surgery	
Laparoscopy	16 (40%)
Laparotomy	24 (60%)
Chemotherapy	
Yes	27 (67.5%)
No	13 (32.5%)
Pregnancy plan	
No	27 (67.5%)
Yes	13 (32.5%)

All patients were regularly followed up as described in Patients and Methods. Median follow up time was 54 months, with range of 25~201 months. The 2 year and 5 year of disease-free survival (DFS) were respectively 95 and 87.5%. 16 (40%) patients received laparoscopic staging surgery, and 2 (12.5%) of them had relapse in 5 years. Meanwhile, 2 in 24 patients (8.3%) who were done surgery through laparotomy relapsed. 67.5% patients received chemotherapy after primary staging surgery. 4 patients (10%) had disease recurrences (Table 2). One patient was upgraded to IIB after laparoscopic comprehensive staging surgery and insisted sparing fertility. The histological type was mucinous and the patient received 4 cycles of paclitaxel plus carboplatin. Unfortunately tumor recurrence happened after 4 months. The rest 3 patients were all FIGO IC disease, with mucinous, clear cell and endometroid respectively. They all received at least 3 cycles chemotherapy after primary surgery. No death occurred.

**Table 2:** 4 patients who developed recurrence

Pt	Age at surgery	FIGO stage	Histology, Grade	Type of surgery	Chemotherapy/cycles	DFS (months)
1	26	IC	Mucinous, G1	Laparotomy	Cyclophosphamide + Cisplatinum/5	144
2	29	IC	Endometriod, G1	Laparotomy	Paclitaxel + Carboplatin/6	20
3	40	IC	Clear cell	Laparoscopy	Carboplatin/3	48
4	33	IIB	Mucinous, G1	Laparoscopy	Paclitaxel + Carboplatin/4	4

Pt: patients; DFS: disease free survival

Because of some private reasons, 27 patients (67.5%) were single or contraceptive. 13 patients (32.5%) desired to have a baby after treatment (Table 3). All of them were FIGO IA or IC epithelial ovarian cancer and had no disease recurrence during the whole follow-up period (range of 33~129 months) (Table 3). 5 patients (38.5%) were infertility. Among them, 3 patients had chemotherapy with paclitaxel plus carboplatin, one with cyclophosphamide plus cisplatin, and the rest one patient didn’t receive any chemotherapy. 8 patients (61.5%) have successfully conceived spontaneously. All of them received common obstetric examination and regular oncologic follow up. Any radiographic test was avoided and CA125 was not regularly detected during pregnancy. The pregnancy outcomes have been shown in Table 4. 3 patients had fetus loss, including 1 natural abortion in the first trimester, 1 fetal anomaly (pregnancy was terminated at 23 weeks) and 1 fetus death (24 weeks) in uterus without exact reasons. 5 patients (38.5%) had live births, including 2 premature births and 3 term births (Table 4). There were no serious adverse in obstetric and neonatal outcomes among women with live births. No tumor recurrence happened during pregnancy.

**Table 3:** Pregnancy outcomes of 13 patients

	N (%)
Age at surgery, years Median (range)	27 (18~40)
Histology	
Serous	1 (7.7%)
Mucinous	7 (53.8%)
Endometriod	3 (23.1%)
Clear cell	2 (15.4%)
FIGO stage	
IA	4 (30.8%)
IC	9 (69.2%)
Chemotherapy	
Paclitaxel + Carboplatin	8 (61.5%)
Cyclophosphamide + Carboplatin	1 (7.7%)
Cyclophosphamide + Cisplatin	2 (15.4%)
No chemotherapy	2 (15.4%)
Pregnancy outcomes	
infertility	5 (38.5%)
Miscarriages	3 (23.0%)
Live birth	5 (38.5%)
Premature birth	2
term birth	3

**Table 4:** 8 Patients with early epithelial ovarian cancer who were pregnancy after staging surgery

Pt	Age at surgery	FIGO stage	Histology, Grade	Type of surgery	Chemotherapy /cycles	^*^Interval	Pregnancy outcome
1	27	IA	Endometriod, G1	Laparotomy	Paclitaxel + Carboplatin/7	36	Miscarriage during 1st trimester
2	30	IC	Mucinous, G1	Laparotomy	Cyclophosphamide + Carboplatin/3	24	Dead fetus in uterus at 24 weeks
3	30	IC	Mucinous, G1	Laparotomy	Paclitaxel + Carboplatin/7	22	Fetal anomaly at 23 weeks
4	28	IA	Mucinous, G1	Laparoscopy	Cyclophosphamide + Cisplatin/3	12	Vaginal delivery at 33 weeks because of PROM
5	33	IC	Mucinous, G1	Laparotomy	Paclitaxel + Carboplatin/6	36	Vaginal delivery at 38 weeks
6	30	IA	Clear cell	Laparotomy	No chemotherapy	12	Vaginal delivery at 39 weeks
7	27	IC	Serous, G1	Laparoscopy	Paclitaxel + Carboplatin/5	1	Cesarean section at 37 weeks
8	26	IC	Mucinous, G1	Laparotomy	Paclitaxel + Carboplatin/6	60	Vaginal delivery at 36 weeks because of spontaneous labor

Pt: patients; ^*^interval: the interval between terminal of chemotherapy with pregnancy diagnosed; PROM: premature rupture of membranes

## Discussion

For young patients with early stage epithelial ovarian cancer, fertility-sparing surgery (FSS) is important. The first reports about FSS for EOC appeared in 1960s [8]. Then many case series undergoing FSS were reported. JCOG-FSS study and JCOG1203 study carried out by the Japan Clinical Oncology Group (JCOG) confirmed that FSS is safe for stage IA clear cell carcinoma (CCC) and IC non-CCC G1/G2 diseases [9, 10]. In 2011, Kajiyama reported that there were no any differences finding in oncologic outcome between radical surgery and FSS in stage I EOC patients. In FSS group, 52 patients (86.7%) survived without relapse. There was no difference in disease free survival (DFS) and overall survival (OS) between stage IC (capsule rupture) and stage IA. The pregnancy rate was 15% (9/60). The authors suggested FSS can be safely proposed to all patients [11]. Many other retrospective analysis supported this conclusion [12, 13]. 2007 guidelines of American College of Obstetrics and Gynecology (ACOG) and 2008 guideline s of the European Society for Medical Oncology (ESMO), FSS was adopted for stage IA EOC patients and non-clear cell histology G1 and G2 [14, 15]. Limited data supported stage IB EOC patients or IC with bilateral ovarian disease preserving a part of the contralateral ovary. National comprehensive cancer network (NCCN) guideline version 2. 2018 of ovarian cancer suggested preserving the uterus only. In this retrospective study, 40 patients were all treated and regularly followed up in one tertiary hospital in Beijing, China. 5 years of DFS was 87.5% which is consistent with other reports. 4 patients relapsed but no death occurred. Based on above, for young EOC patients who desired to preserve fertility, FSS could be suggested in clinic. Besides, through a prospective study published in 2016, 6.1% EOC patients were reclassified to a higher stage after laparoscopic fertility-sparing surgery. The overall survival of median follow-up period 38 months was 95.4% and recurrence-free survival 84.6% [16]. This work also showed 6.25% patients with laparoscopic staging surgery and 4.2% with laparotomy staging surgery were upstaged. Laparoscopic staging surgery may a viable option for early stage EOC patients.

Except oncologic prognosis, pregnancy outcomes were also focused in this work. Marriage and reproduction of offspring are both not only individual behaviors but also social behaviors. 27 in 40 patients (67.5%) who desired to preserve fertility at the beginning of cancer treatments were still single or have given up pregnancy intent. 5 in 13 patients who had baby plan had live births. The fertility rate was consistent with other published studies [17, 18]. In 5 patients with live birth in this study, Platinum plus paclitaxel were used in 3 patients after surgery. 1 patient received 3 cycles of Cyclophosphamide plus carboplatin and delivered at 33 weeks because of premature rupture of fetal membranes. 1 patients was diagnosed with pregnancy only 1 month after the end of chemo-therapy and no neonatal malformation was found. All patients during pregnancy received common obstetric management and oncological follow-up. More appropriate medical managements for EOC patients after FSS and chemo-therapy during pregnancy has not been built up because of limited experience. There were no adequate evidence shown that platinum plus paclitaxel could lead to fetal mortality, abortion and neonatal malformation [19]. However, some other chemotherapeutic agents, such as alkylating, were proved poisonous to ovary which might result in premature ovarian failure and subsequent infertility [20].

This was a retrospective study in one tertiary hospital of China. Compared with multicenter research, the deviation and bias of data might be best eliminated. Nearly 18 years ago, fertility-staging surgery was gradually carried out in stage IA/IC low grade EOC patients who desired conserving fertility in Peking Union Medical College Hospital. All patients received complete staging surgery through laparoscopy or laparotomy. Chemo-therapy was varied according to patients’ conditions. And optimistic oncologic outcome was gained. Our experience supported us continuing this work for early stage EOC patients. The pregnancy outcomes for them was summarized for the first time. The rate of live birth was low but close to other published studies in the world. The sample was too small to analysis the high risk factors of infertility and abnormal pregnancy. And the follow-up time was so short for some absolutely young patients who have not been married yet. The pregnancy rate might be improved if the following-up could be continued.

## Abbreviations

BSO: Bilateral salpingo-oophorectomy
CCC: Clear cell carcinoma
DFS: Disease free survival
EOC: Epithelial ovarian cancer
FSS: Fertility sparing surgery
OS: Overall survival
TAH: Total abdominal hysterectomy
USO: Unilateral salpingo-oophorectomy
